# Constructing fine-grained entity recognition corpora based on clinical records of traditional Chinese medicine

**DOI:** 10.1186/s12911-020-1079-2

**Published:** 2020-04-06

**Authors:** Tingting Zhang, Yaqiang Wang, Xiaofeng Wang, Yafei Yang, Ying Ye

**Affiliations:** 1 Basic Medical School, Chengdu University of Traditional Chinese Medicine, No. 37, Shi Er Qiao Road, Chengdu, 610075 People’s Republic of China; 2 College of Software Engineering, Chengdu University of Information Technology, No. 24, Xue Fu Road, Chengdu, 610225 People’s Republic of China

**Keywords:** TCM clinical records, Fine-grained annotation, Named entity recognition, Corpus construction, Guideline development

## Abstract

**Background:**

In this study, we focus on building a fine-grained entity annotation corpus with the corresponding annotation guideline of traditional Chinese medicine (TCM) clinical records. Our aim is to provide a basis for the fine-grained corpus construction of TCM clinical records in future.

**Methods:**

We developed a four-step approach that is suitable for the construction of TCM medical records in our corpus. First, we determined the entity types included in this study through sample annotation. Then, we drafted a fine-grained annotation guideline by summarizing the characteristics of the dataset and referring to some existing guidelines. We iteratively updated the guidelines until the inter-annotator agreement (IAA) exceeded a Cohen’s kappa value of 0.9. Comprehensive annotations were performed while keeping the IAA value above 0.9.

**Results:**

We annotated the 10,197 clinical records in five rounds. Four entity categories involving 13 entity types were employed. The final fine-grained annotated entity corpus consists of 1104 entities and 67,799 tokens. The final IAAs are 0.936 on average (for three annotators), indicating that the fine-grained entity recognition corpus is of high quality.

**Conclusions:**

These results will provide a foundation for future research on corpus construction and named entity recognition tasks in the TCM clinical domain.

## Background

Chinese electronic medical records (EMRs) contain much information about clinical diagnoses and treatment events. Since the publication of the *Basic Norms of Electronic Medical Records* in China, a solid body of data has been generated as a result of the unprecedented expansion of EMRs. Traditional Chinese medicine (TCM) is a unique and complicated medical system that has been developed over thousands of years [1]. It is becoming a complementary and alternative medical system in Western countries. Although the Chinese Ministry of Health has issued a series of relevant regulations [2], TCM records contain a large amount of clinical information, such as the chief complaint, four diagnoses, and treatment measures, stored as unstructured data in the clinical narrative. Unfortunately, these unstructured data are difficult to use directly in clinical practice.

With the continuous development of information technology, much of the knowledge contained in large-scale TCM clinical records has been increasingly mined for data-driven medical studies, clinical decision making, and health management. Natural language processing (NLP) techniques, which assist the automatic processing and analysis of EMRs, have become increasingly used in the field of TCM analysis in recent years [3]. Named entity recognition (NER) [4, 5] is a high-level task in NLP, and a human-annotated entity corpus is an indispensable resource for training automated NER systems and testing their performance. In English, some medical knowledge bases, such as terminology systems like the Unified Medical Language System [6], clinical ontology systems like the Systematized Nomenclature of Medicine-Clinical Terms (SNOMED CT) [7], and medical databases like DrugBank [8], contribute to NER in clinical records. In China, some resources have been developed for NER tasks in the Chinese clinical domain; for example, the Traditional Chinese Medicine Language System (TCMLS) standardizes the terminology of TCM. Currently, some entity types in Chinese clinical records, such as medications, anatomy, treatments, tests, symptoms, body parts, temporal words, drugs, and operations [5, 9–13] have already been annotated. However, to the best of our knowledge, open Chinese annotated corpora rarely include TCM clinical records. The lack of TCM clinical datasets is partly due to concerns regarding patients’ privacy as well as concerns about revealing unfavorable institutional practices [14], so these records are very private and scarce; another reason is the high complexity of Chinese clinical text analysis. This type of text has sublanguage features [15], so the characteristics of raw TCM free-text clinical records are very different from the characteristics of common texts in the Chinese language. For instance, the text has a narrative form, uses a concise style similar to classical Chinese, and employs nonstandard descriptions [16]. Hence, constructing a corpus of TCM clinical records remains difficult, and the electronic capture or retrieval of TCM clinical text data has been a challenge; thus, research into NLP tasks on TCM clinical free text is still at a preliminary stage.

Currently, most of the relevant studies, such as [5, 10, 11], do not present a standardized process-based approach to the construction of a corpus, especially in the steps of data selection, guideline drafting, and annotation. To date, there is no existing fine-grained annotation schema applicable to the TCM clinical domain. Hence, this study focuses on a fine-grained corpus construction method that is suitable for the clinical free text of TCM. On the basis of existing approaches, we propose a four-step method to make the entire process clear, replicable, and consistent. Fine-grained annotation guidelines for TCM clinical text were also developed. The statistical analysis indicates that the method and guidelines are appropriate and effective. The results of this study will provide a foundation for future research into corpus construction and effective NER tasks in the TCM clinical domain.

## Related work

In recent years, research on clinical EMRs has become a popular topic [17]. Studies on English EMR entity corpora began early, and text mining and NLP applications, algorithms, and corpora in the English language are relatively mature. There are some well-known publicly available annotated corpora, such as GENIA (Genome Information Acquisition) [18] for data mining and information extraction in the molecular biology domain, NCBI (National Center for Biotechnology Information) Disease [19] for disease names and adverse effects, and drug-drug interactions (DDI) [20] for pharmacological substances and drug interactions. Moreover, the integrating biology and the bedside (i2b2) challenges have contributed to clinical NLP studies. For instance, i2b2 organized challenges on the extraction of medical information from English discharge summaries in 2009 and 2010: the concepts of extraction involve drugs, doses, duration, medical problems, treatment, and testing. Since 2006, i2b2 has released nine corpora for evaluating EMR information extraction. Based on these corpora, great strides have been made in NER research on English discharge summaries. The annotation schemes and evaluation methods of corpus construction in English possess high reference value for Chinese clinical notes. Despite this, the development of corpus construction in Chinese medicine has fallen behind that of English Western medicine, and the availability of large corpora in Chinese is currently limited. Influenced by the rapidly developing English medical corpora, the drive to construct Chinese medical corpora has gradually begun to move forward. The major annotated corpora of Chinese medical notes are summarized in Table 1 and described in detail in the following section.

**Table 1:** Studies on the construction of Chinese clinical text corpora in the last five years

Year	Author	Scale and target	Entities	Fine-grained	TCM clinical texts
2014	Xu et al. [9]	336 Chinese discharge summaries of 71,355 words	Medication, anatomy, medical problems, treatments, and tests	N	N
2014	Lei et al. [5]	400 admission notes and 400 discharge summaries	Clinical problems, procedures, laboratory tests, and medications	N	N
2014	Wang et al. [21]	11,613 clinical records	Symptoms	N	Y
2014	Wang et al. [22]	115 EMRs	115 documents on tumor-related information from the notes of hepatic carcinoma operations	N	N
2014	Gao et al. [23]	42 health records of stroke	Body structures and clinical description	N	Y
2015	Li et al. [24]	700 initial diagnosis records, congestive heart failure data of 253 cases.	TCM herbs and symptoms	N	Y
2015	Xu et al. [25]	24,817 anonymized Chinese EMRs	Symptoms, clinical tests, diseases, drugs, body parts, and procedure categories	N	Y
2016	Zhang et al. [26]	2000 notes (1000 admission notes and 1000 discharge summaries)	Diseases and syndromes, symptoms and signs, treatments and drugs, and laboratory tests	N	N
2016	Wan et al. [27]	More than 100,000 TCM article abstracts	Herbs, syndromes, diseases, and formulas	N	Y
2016	Liu et al. [13]	1778 clinical notes of 281 hospitalized patients	Temporal expression and normalization in Chinese clinical notes (type, value, and modifier)	N	N
2017	Ruan et al. [28]	1000 EMRs	Symptoms, departments, diseases, medicines, and examinations	N	Y
2017	He et al. [10]	500 discharge summaries and 492 progress notes	Diseases, symptoms, and treatments	N	N
2018	Zhang et al. [29]	400 documents	Symptoms, tests, diagnoses, treatments, and body parts	N	N
2018	Miao et al. [30]	540 reports	Breast Imaging Reporting and Data System	N	N
2018	Bao et al. [31]	600 documents	History of present illnesses, personal history, and family history	N	N
2019	Wang et al. [32]	1596 annotated instances (10,024 sentences)	Diseases, symptoms, exams, treatments, and body parts	N	N
2019	Gao et al. [11]	255 authentic admission records	Medical discovery, body parts, temporal words, diseases, medications, treatments, inspections, laboratory tests, and measurements	N	N
2019	Cai et al. [12]	1000 admission records	Anatomical parts, symptom descriptions, independent symptoms, drugs, and operations	N	N
2019	Xiong et al. [33]	1000 admission notes and 800 discharge summaries	Body parts, diseases, symptoms, tests, and treatments	Y	N

### Chinese clinical entity recognition corpus construction

Based on the concept annotation guidelines from the 2010 i2b2 challenge, Xu et al. [9] labeled a standard corpus of 336 Chinese discharge summaries (medications, anatomy, medical problems, treatments, and tests) in 2014. The annotation work consisted of two rounds; the first round was completed by three annotators with the relevant domain background, and the second round was conducted by three annotators with backgrounds in computer linguistics. The results were refined and the final gold standard was obtained by combining the results of the first two rounds. Lei et al. [5] constructed an annotated entity corpus of 400 discharge summaries and 400 admission notes. The guidelines were similar to those used in the 2010 i2b2 NLP challenge, but the “treatments” were divided into “procedures” and “medications.” Moreover, Wang et al. [22] annotated the text with 12 elements required by doctors from free-text operation notes. In this study, the guidelines are not mentioned and the annotation process is briefly described. Miao et al. [30] annotated Breast Imaging Reporting and Data System categories manually in a preliminary study on information extraction from Chinese breast ultrasound reports. These two studies [21, 29] are good examples of information extraction for specific information. Liu et al. [13] annotated temporal expressions in clinical notes and built guidelines that refer to the temporal expression annotation guidelines of TimeML for English newswire text and the 2012 i2b2 NLP challenge for English clinical text. Furthermore, in 2019, Gao et al. [11] described a more detailed method of constructing a corpus of nine entity types based on resident admit notes. The guideline was also developed using the i2b2 annotation guidelines, but they added the “body part” and “temporal word” entities in their annotation work, and the “inspection” and “laboratory test” entities are distinguished. An iterative annotation method was employed to form the manual annotation scheme. Furthermore, He et al. [10] used an annotation method for English clinical text to build a syntactic corpus about entity diseases, symptoms, and treatments. They created the draft guideline first, then trained the annotators and updated the guideline. The inter-annotator agreement (IAA) was then calculated to measure the quality of annotator training, and they constructed the corpus. The method used in this study is a good demonstration of the construction of a Chinese clinical corpus; however, similar to previous studies, it adopts coarse-grained tagging patterns. Encouragingly, in 2019, Xiong et al. [33] manually annotated a corpus with Chinese word segmentation and part-of-speech tags for Chinese clinical text at a fine granularity. This work is an excellent reference, but it does not elaborate on the methods and steps used in the annotation. In summary, there has been some excellent initial progress toward the construction of Chinese clinical record corpora. However, there is still no standardized methodology for Chinese clinical text.

### TCM corpus construction

**Fig. 1:**
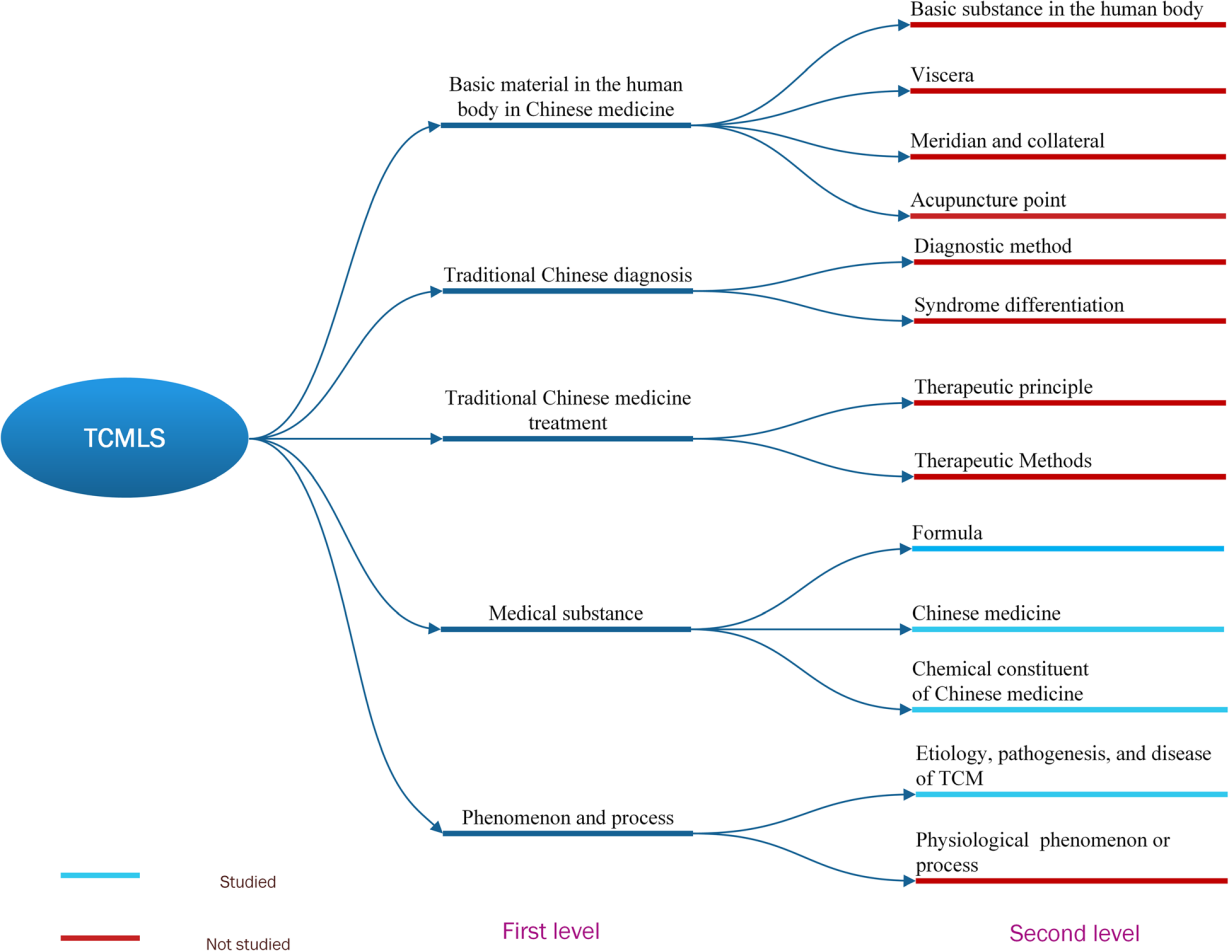
First and second levels of concepts in TCMLS. The figure was generated by Mcrosoft Visio 2013

In contrast to the progress in the corpora of Western medicine in Chinese medical records, the progress on corpora of TCM clinical notes is still in its infancy. Fang et al. [34] annotated a large biomedical literature corpus obtained from PubMed and developed an open database, TCMGeneDIT, to provide information about TCM, genes, diseases, TCM effects, and TCM ingredients. However, this research approach cannot be used for clinical records. With respect to clinical records, Wang et al. [21] constructed an annotated corpus for the symptoms of the chief complaint in TCM-free text. It is an empirical study, but the number of text types and entity categories in the corpus was relatively small, and the authors did not list detailed methods about the development of their guidelines and annotation. Ruan et al. [28] focused on symptoms and symptom-related entity extraction. Symptoms were divided into TCM symptoms and Western symptoms, and medicine was divided into TCM medicine and Western medicine. The dataset was divided into two parts; two experts annotated the symptom entities in the EMRs to train and test a conditional random field (CRF) model. Li et al. [24] built a dataset of herbs and symptom records and annotated the relationships between them. This study is useful for the extraction of relations from TCM health records. Fig. 1 presents an overview of the concepts found in TCM clinical notes, e.g., meridians and collaterals, viscera, acupoints, the etiology of TCM, syndrome elements, and diagnosis methods. These concepts were not all addressed in previous studies.

These studies demonstrate that the research on TCM clinical text has some defects: 1) there are no large corpora available in the TCM domain; 2) only a small part of the overall TCM concepts have been annotated systematically, while other types of entities have been ignored; 3) the existing methods of TCM corpus construction are too coarse grained; and 4) most of the previous studies do not describe how data selection, guideline drafting, and annotation were implemented. Hence, a practical and effective method is needed to develop a standard annotation scheme and build a comprehensive entity corpus of TCM records.

## Methods

### Dataset

The dataset contains 10,197 records, which are fragments extracted from a modern Chinese TCM case records database. These records are transcripts of raw TCM clinical records collected by TCM doctors during their routine diagnosis and treatment work. Our dataset does not contain basic information about patients, such as name, age, or gender. The reason for this is two-fold:

1. The transcripts of TCM case records are an important resource for studying TCM. A complete case record of TCM contains abundant TCM knowledge, such as the main complaint, syndrome differentiation, diagnosis, treatment or prescription, medicines, and doses. Therefore, it is an important resource of medical information for the study of unstructured documents, and is the best type of document for obtaining the analysis and clinical experience of well-known TCM experts [35–37]. In contrast to resident admit notes, the TCM case records are more refined, logical, and enlightening [38]. One example is the text “咳嗽,黄稠痰,痰不易吐出,咽喉疼痛,咳引两太阳穴痛,怕冷,无汗,口气秽臭,苔黄腻,舌略红,脉不浮” (cough, the yellow thick phlegm is not easy to spit out, throat pain, traction pain in the position of EX-HN5 when coughing, fear of the cold, no sweat, fetid breath, yellow and greasy coating, slightly red tongue, pulse is not floating), in which the key symptoms for the TCM diagnosis have been listed. These were obtained by the four basic diagnosis procedures (inspection, listening and smelling, inquiry, and palpation) [39].
2. The extraction of knowledge hidden in a large number of TCM clinical texts and distillation of this knowledge into a concise form is clinically significant. A good example is the discovery of artemisinin, which was spotted in TCM records. The use of artemisinin is a medical advance that has saved millions of lives globally [40]. More recently, many studies have increasingly found that the diagnostic methods of TCM can help the diagnosis of disease in modern medicine. For example, it was found that tongue features can be used to predict early-stage breast cancer [41, 42]. Moreover, a geographical tongue^1^ is associated with the severity of diseases such as psoriasis [43]. With regard to pulse diagnosis, Wang et al. [44] found that there is a significant difference between the pulse signals of healthy volunteers and patients with fatty liver disease and cirrhosis. In TCM a “string-like” pulse^2^ in the left hand is closely related with liver disease [45].

For these reasons, we employed parts of TCM transcript data to design a feasible and reusable method for establishing a fine-grained entity corpus of TCM clinical records. We note that because personal patient information is not included in the dataset, the study requires no ethics committee approval.

### Entity selection

The method used to select entities is rarely mentioned in previous studies. In our work, we combine sample annotation with repeated discussions. First, we analyzed the characteristics of our dataset. Then, 100 randomly selected records were given to each annotator to establish the entity labels and annotate the records. After this step, 26, 10, and 46 concepts were marked by each of the three annotators. The three annotators discussed the inconsistent labels to reach a consensus about which entity types should be included in our study. The annotators’ understanding of four entity categories (“body parts,” “tongue diagnosis,”^3^ “pulse diagnosis,”^4^ and “direction and position”) was more consistent than their understanding of the others. To improve the work efficiency and quality, we chose these four entity categories rather than all the categories of TCM entities that occur in the dataset. There are some important concepts not involved in our study, for example, “symptoms,” “temporal words,” and “herbal medicine,” that we plan to address in future research.

The four categories in our experiment, which consist of 13 entities, are highly important to pathogenesis analysis, syndrome differentiation, diagnosis, and treatment. For example, in the phrase “疏肝利胆” (dispersing stagnated liver qi for promoting bile flow), “肝” (the liver, a “Zang organ” entity) and “胆”(the gallbladder, a “Fu organ” entity) reflect the key Zang-Fu organs^5^ in the treatment procedure. In the Chinese word “肩髃痛” (pain at LI15), “肩髃”(LI15, an “acupoint” entity) indicates that the pathogenesis is an abnormality of the meridian qi of the large intestine meridian (LI). Moreover, as an *ashi* acupoint,^6^ “肩髃” (LI15) has a good curative effect for shoulder pain. Moreover, pulse diagnosis and tongue diagnosis are indispensable in TCM. For instance, when a particular pulse appears at the wrong place or in the wrong season, a serious disequilibrium of the system is indicated [46]. Furthermore, the tongue body mainly reflects a deficiency or excess of qi and blood in the Zang-Fu organs, whereas a change in the tongue coating is mainly used to judge the depth and severity of pathogenic qi [47]. For example, in the transcript “失眠,口苦,思饮,咯痰略黄,大便偏干,鼻息热,眼干,苔黄干,脉细弱” (insomnia, bitter taste, fond of drink, slightly yellow sputum, dry stool, hot breath, dryness of eyes, yellow and dry coating, thready and weak pulse), the “yellow and dry coating” reflects the internal disturbance of pathogenic heat, and the “thready and weak pulse” indicates the deficiency of healthy qi. Furthermore, position and direction have significant clinical diagnostic value. For example, according to TCM theory, different positions of the tongue correspond to five different Zang organs: the top of the tongue corresponds to the heart, so “舌尖红” (red tip of the tongue) is probably a manifestation of heart fire.

### Entity definition

In this study, we summarized four data categories, and 13 entity types are derived from these four categories. Referring to the concept definitions of TCM in WHO’s international standard terminologies on traditional medicine in the Western Pacific region [48] and a text book on the diagnostics of TCM [47], the definitions of 13 entities are listed in Table 2. More details and examples are shown in the guidelines in Additional file 1.

**Table 2:** Definition and examples of the 13 entities used in this study

Entity type	Definition	Examples (entities are in bold font)
**Ordinary body part**	This entity enables us to locate the exact positions of symptoms, medical tests, or disease.	**眼**痒 (itchiness in the eyes)
**Tongue body**	This is the musculature and vascular tissue of the tongue, also the tongue substance. It is annotated only when followed by a specific description of the tongue’s physical manifestation.	**舌**红, 苔黄, 脉滑 (red tongue, yellow coating, slippery pulse)
**Tongue coating**	A layer of moss-like material covering the tongue, also called tongue fur. It is annotated only when followed by the description of tongue coating manifestation.	舌红, **苔**黄, 脉滑 (red tongue, yellow coating, slippery pulse)
**Pulse**	A radial artery of the wrist, which includes three sections: *cun*, *guan*, and *chi*. The pulse entity is annotated only when it is followed by a description of the pulse condition.	舌红, 苔黄, **脉**滑 (red tongue, yellow coating, slippery pulse)
**Acupoint**	A point where a needle is inserted and manipulated in acupuncture therapy.	**肩髃**痛 (pain in LI15)
**Meridian and collateral**	A system of conduits through which qi and blood circulate, connecting the bowels, viscera, extremities, superficial organs, and tissues, and making the body an organic whole. These are the same as channels and networks and are also called meridians or channels.	左大腿**阳明经**固定痛 (fixed pain in the stomach channel of the foot-*yangming* of the left leg)
**Zang organ**	An internal organ in which the essence and qi are formed and stored. These organs include heart, liver, spleen, lungs, and kidneys, and are also called the five viscera.	一直服调**脾**化湿药 (always take the medicine for regulating the spleen and removing dampness)
**Fu organ**	An internal organ in which food is received, transported, and digested, including the gallbladder, stomach, large intestine, small intestine, urinary bladder, and triple energizers.^g^ They are also called the six bowels.	
**Both the tongue body and tongue coating**	Words referring to the tongue body and tongue coating.	**舌**可 (normal tongue)
**Tongue body manifestation**	Specific description of the tongue body manifestation, including tongue color, shape, and sublingual vein.	舌**红**, 苔黄, 脉滑 (red tongue, yellow coating, slippery pulse)
**Tongue coating manifestation**	Specific tongue coating manifestation, including color, thickness, and texture.	舌红, 苔**黄**, 脉滑 (red tongue, yellow coating, slippery pulse)
**Pulse condition**	Specific description of arterial pulsation in TCM when the pulse is felt during examination.	舌红, 苔黄, 脉**滑** (red tongue, yellow coating, slippery pulse)
**Direction and position**	Description of the direction and position, which enables us to know the specific location of the body part.	**左**膝关节疼痛 (pain in the left knee joint)

^g^In TCM, the Fu organ, or “triple energizers,” is a collective term for the three portions of the body cavity through which the visceral qi is transformed. This organ is also widely known as the “triple burners.” It contains the upper energizer, middle energizer, and lower energizer. It is also called the “solitary hollow organ,” because there is no paired relationship between the viscera and the “triple energizers”

### Annotation tools

**Fig. 2:**
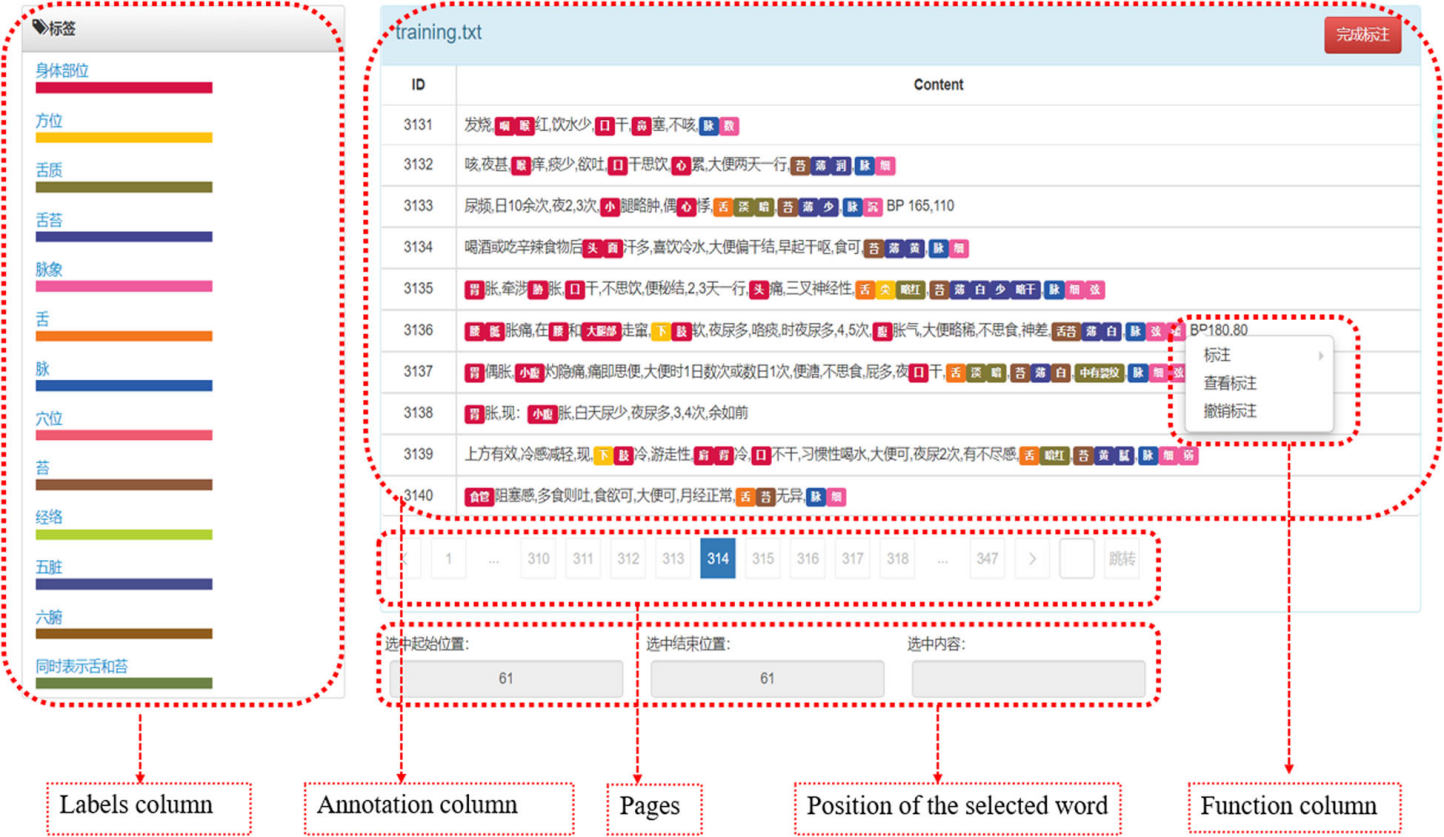
Screenshot of the entity annotation tool

To make the fine-grained marking process easier and more efficient, we developed an entity annotation tool. As shown in Fig. 2, the Chinese characters were labeled with predefined tags with a specific color. By specifying the color of the label, we can distinguish the content of continuous annotations and make inconsistencies more visible. This will facilitate the modification of the annotations and the recording of the problems. With this annotation tool, annotators are able to add and remove labels in the labels column, remove incorrect annotations and re-annotate them in the function column, and annotate entities in the annotation column. The location information of the selected content is displayed in the position column.

### Fine-grained annotation

**Fig. 3:**
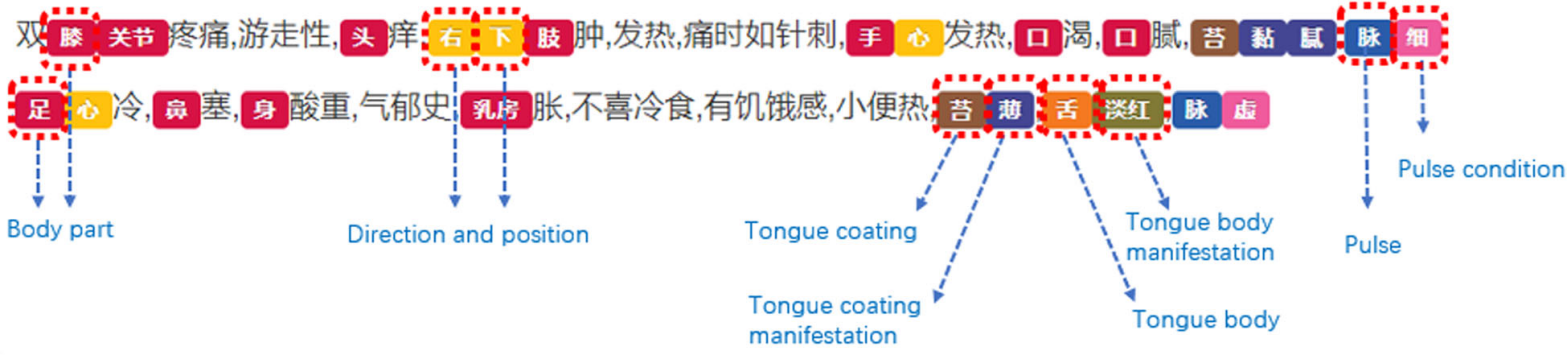
Examples of fine-grained annotation

Fine-grained annotation further divides the coarse-grained entities into finer subcategories until no further divisions can be made, and the Chinese words are then further divided into the smallest semantic units. Consequently, most of the words are shorter than two Chinese characters. In this way, more context information can be captured. Therefore, a fine-grained annotated corpus will better support the automatic processing and analysis of EMRs in NER. For instance, Roberts et al. [49] determined that high-quality fine-grained natural language annotations substantially affect a system’s ability to recognize heart disease risk factors. However, most of the studies on Chinese clinical entity tagging in recent years employ a coarser-grained annotation, for example, “右下肢” (the right lower limb) was annotated as a “body part.” In contrast, in our fine-grained annotation guidelines, as shown in Fig. 3, “肢” (a limb) should be annotated as an “ordinary body part,” and “右” (right) and “下” (lower) should be separately annotated as “direction and position.”

### Annotation method

**Fig. 4:**
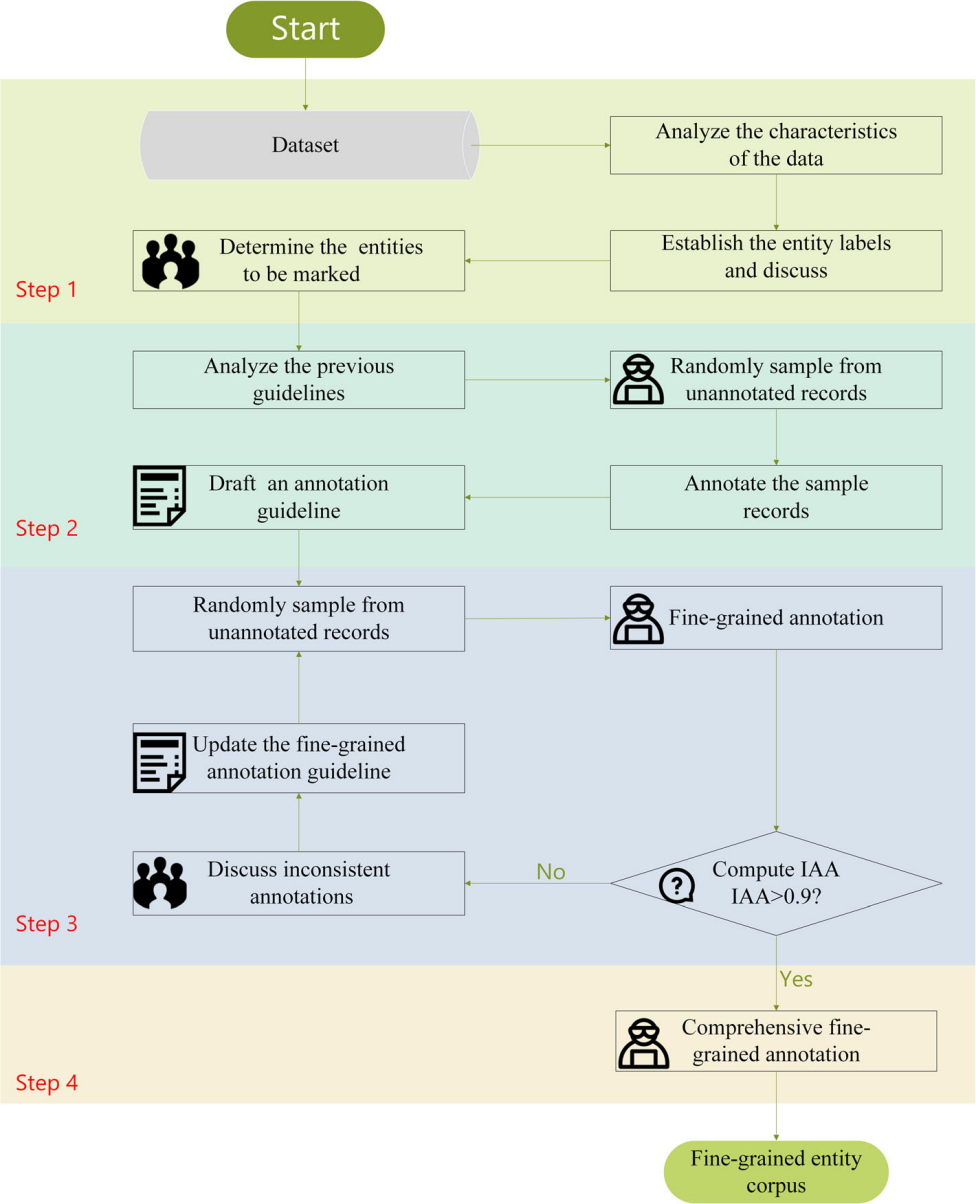
Workflow of entity selection, guideline development and corpus construction, and IAA measurement. The figure was generated by Mcrosoft Visio 2013

Using previous research methods as references, we designed a replicable method to develop a fine-grained annotation guideline and construct a fine-grained entity corpus. The approach consisted of the following four steps (Fig. 4).

1. Determination of the entities to be marked (as described in detail in Section 3.2).
2. Guideline drafting: After referring to some existing well-developed guidelines [26, 50, 51], a team of three annotators randomly selected 300 records (100 each) from the dataset and independently annotated the samples. At the same time, they summarized the characteristics of the included entities and continually discussed them. Finally, a fine-grained annotation guideline was drafted in which examples of different cases were included for easier understanding.
3. Guideline updating and consistency assessment: In each round, 100 unannotated records were randomly selected from the dataset. The guideline was constantly updated until the IAA met the standard of satisfaction (κ > 0.9) which meant the labels of the three annotators were highly consistent. Otherwise, the iterative fine-grained annotations on the sample records were continued. During this step, we added more examples and supplemented the draft guidelines with detailed explanations. A more comprehensive set of fine-grained annotation guidelines was hence developed (see Additional file 1).
4. Corpus construction: Using the guidelines developed in steps 2 and 3, three annotators performed the annotation work independently. The dataset was divided into three parts, and the three annotators marked different parts separately to reduce the time required and improve annotation efficiency. During this period, we kept the annotation work as independent as possible, and the following principles were strictly followed: i) Although there are practical standards for medical record writing, sometimes errors exist in these texts. Incorrectly written characters were not annotated in any situation. For example, in the word “脚指” (foot finger), “指” (finger) is miswritten and should be “趾” (toes). Hence, “指” (finger) was not annotated. ii) Punctuation should not be included in the annotation as much as possible. This is to minimize the interference of punctuation on the annotated entities. iii) Entity annotation can be nested but not overlapped. For example, “指掌连接处” (the body part where the fingers and palms are connected) should be annotated as an “ordinary body part” but “指” (finger) and “掌” (palm) should also be annotated as “ordinary body parts” individually. iv) For some complex or ambiguous situations, the annotators discussed how to unify the decisions. For example, there was some controversy as to whether “心” (heart) in the word “心悸” (palpitation) should be annotated as an “ordinary body part” or a “Zang organ.” Here, “心悸” (palpitation) is a subjective sensation of the rapid and forceful beating of the heart. It seems logical to annotate “心” (heart) as both an “ordinary body part” or a “Zang organ.” After discussions, and considering that “心悸” has been a symptom name in TCM for more than a thousand years, the annotators formed a consistent view; that is, “心” (heart) in such situations should to be consistently annotated as a “Zang organ.”

In addition, during the comprehensive fine-grained annotation process, some measures were taken to ensure the quality: 1) Annotators were required to record uncertain annotations, and they discussed them regularly until all the ambiguities were resolved. 2) Three annotators with similar TCM backgrounds (with doctor qualifications in TCM and in the same research area) improved the marking accuracy and reduced the occurrence of uncertain cases. 3) Duplicate documents were assigned to three groups in step 4 for an IAA evaluation in order to ensure the quality of the annotated data.

### Key and difficult points in the entity annotation task

Our study is the first to use fine-grained annotation methods in the TCM clinical records. Entities such as “acupoints,” “Zang-Fu organs,” “tongue manifestations,” and “pulse conditions” have not been annotated in previous studies. Consequently, the annotation work is challenging. The key points and difficulties are as follows.

Clinical narratives are often written in a medical sub-language with semantic categorization of words, domain specific terminology, incomplete phrases, and omission of information [52]. TCM case records are written by practicing doctors, and their brief forms appear very similar to ancient Chinese texts. Moreover, they can only be understood by a professional doctor with a background in TCM. For example, in the transcripts “痛点, 左右耳门” (pain point, at left and right TE21), “耳门” (TE21) is an acupoint other than ordinary body part. In another example, “颈项” (neck), “颈” (front of the neck), and “项” (back of the neck) are entities of ordinary body parts.

Fine-grained annotation is the most important and difficult part of our work. One major difference between Chinese and English text is that words in Chinese are formed by continuous Chinese characters without any spaces, and the boundary between fine-grained entities is not clear (as shown in Fig. 3); as a result, fine-grained annotation on TCM clinical notes is time-consuming and annotators must have an in-depth understanding of the document.

### IAA

The calculation of IAA (often known outside of corpus linguistics as the inter-rater agreement) is motivated by the need to address the problem of subjectivity in judgments about things that are not observable with the senses [53]. In our study, we choose Cohen’s kappa to measure the consistency of the three annotators’ work. Cohen’s kappa is a coefficient of internal consistency and is a widely used index for assessing IAA. It is appropriate for nominal and ordinal data when there are two or more raters per subject and is calculated as follows [54, 55].

$$ \upkappa =\frac{P_0+{P}_e}{1-{P}_e} $$

Here, *P*_*0*_ is the observed agreement between two annotators, and *P*_*e*_ is the probability of agreement between the annotators if each annotator were to randomly pick a category for each annotation. It is computed from a contingency matrix representing agreements and disagreements. The annotation is considered to be sufficiently consistent when all three κ values are greater than 0.9.

## Results and discussions

### Annotation consistency

**Fig. 5:**
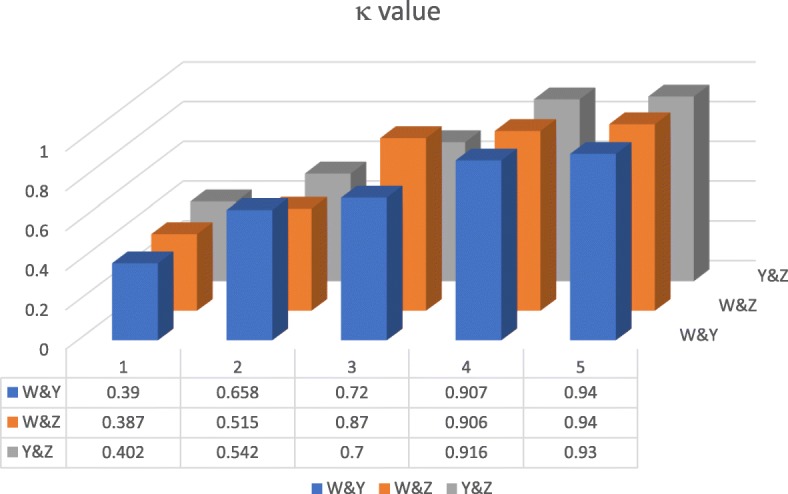
Pairwise IAA (κ) of the three annotators (W, Y, Z) for the first four rounds of annotation and final corpus construction. The figure was generated by Mcrosoft Word 2016

We added duplicates (600 records) to each annotator’s tagging task to calculate the IAA. The result shows that the IAA value during corpus construction remained at a relatively high level (0.93, 0.94, and 0.94; Fig. 5). The IAA evaluation shows that this fine-grained entity corpus is of good quality.

As shown in Fig. 5, our marking task was repetitive and time-consuming work, in which the whole marking process took five rounds to complete. In the fourth round, the IAA values exceeded 0.9, indicating that the three annotators had a high degree of consistency in the understanding of labels and TCM records, and they had ability to accomplish these annotation tasks with satisfactory consistency. As shown in Fig. 5, the IAA values in each annotation round are higher than those of the previous round, showing that our method of iterative annotations and discussions is effective.

### Data analysis of annotations

The fine-grained annotated corpus has 1104 entities and 67,799 tokens in total. An analysis of the corpus reveals some interesting points, especially in terms of the language expressions used in clinical TCM. The data analysis is helpful for identifying the rules of TCM clinical expressions and leads to questions that will contribute to future research about the corpus construction of TCM clinical records.

#### Distribution and analysis of entities and tokens

The distribution of entities and tokens are shown in the Table 3. The proportion of the entities of “ordinary body part,” “tongue body,” “tongue coating,” “tongue body manifestation,” “tongue coating manifestation,” “pulse,” “pulse condition,” and “direction and position” are much higher than those of other entities. In the “body part” category, the entity “ordinary body part” (21,093) occurs the most, followed by the entities “pulse” (6148), “tongue coating” (4978), and “tongue body” (3789). Among the “ordinary body part” entities, we noticed that many annotated entities are concepts from Western medicine. For instance, “毛细血管” (capillary vessel) and “椎间盘” (intervertebral disk) are body part concepts in Western medical anatomy. Clearly, the modern case records of TCM contain both TCM and Western medicine knowledge. In addition, entities related to “tongue body manifestation” (4088), “tongue coating manifestation” (10,911), and “pulse condition” (9573) are relatively common. After reading the original text, we observed that almost every TCM case record documents the pulse or tongue diagnosis information. It can be seen that tongue diagnosis and pulse diagnosis are one of the most common diagnostic methods in TCM, and the “tongue coating manifestation” (10,911) has high diagnostic value in practice.

**Table 3:** Numbers of entities and annotations

Entity classification	Entity type	Total entity count	Total annotation count	Percentage of the corresponding type (entity/annotation)
**Body part**	Ordinary body part	462	21,093	75.3%/56.3%
	Pulse	22	6148	3.6%/16.4%
	Tongue coating	10	4978	1.6%/13.3%
	Tongue body	7	3789	1.1%/10.1%
	Acupoint	87	469	14.2%/1.3%
	Zang organ	5	139	0.8%/0.4%
	Meridian and collateral	16	34	0.98%/0.1%
	Fu organ	2	3	0.3%/0.008%
	Both tongue body and coating	2	793	0.3%/2.1%
	**Total**	**613**	**37,446**	**100%/100%**
**Tongue manifestation**	Tongue coating manifestation	102	10,911	38.9%/72.7%
	Tongue body manifestation	160	4088	61.1%/27.2%
	**Total**	**262**	**14,999**	**100%/100%**
**Pulse condition**	Pulse condition	90	9573	100%/100%
**Direction and position**	Direction and position	139	5781	100%/100%
**Total count**	**13**	**1104**	**67,799**	

As for the distribution of tokens, examples of the top-10 entities in each entity type are shown in Table 4. Combined with the original text, we analyzed the distribution of tag content and revealed that the expressions of many concepts of TCM are not uniform, and there are many entities that are similar in semantics but different in name, e.g., “腹” and “腹部” both mean abdomen, “足阳明经” and “胃经” both refer to the stomach meridian, “内” and “内侧” both mean “inside,” and “中心” and “中间” both refer to the center position. Such synonyms with different expressions on the one hand reduce the reliability of statistical analysis results of the corpus, but on the other hand, are expressions found in real and raw Chinese language data, and identifying them will increase the adaptability of machine learning models. The normalization of these entities will be a part of future research work.

**Table 4:** Examples of the top-10 entities for each entity class

Entity class	Total count	Entity examples (top 10) and number of occurrences
Ordinary body part	21,091	口 (mouth; 2252), 头 (head; 1853), 腹 (abdomen; 1689), 胃 (stomach; 1267), 喉 (larynx; 962), 腰 (waist; 893), 肢 (limbs; 686), 背 (back; 585), 身 (body; 583), 手 (hand; 578)
Pulse	6148	脉 (pulse; 6091), 尺脉 (chi pulse; 11), 肾脉 (kidney pulse; 10), 关 (guan; 6), 寸 (cun; 6), 尺 (chi; 4), 关脉 (guan pulse; 4), 肝 (liver; 2), 沉取 (taking deeply; 1), 脉沉取 (taking the deep pulse; 1)
Tongue coating	4978	苔 (coating; 4765), 舌苔 (tongue coating; 188), 舌 (tongue; 16)
Tongue body	3789	舌 (tongue; 3695), 舌质 (tongue body; 87), 苔 (tongue coating; 3), 舌苔 (tongue coating; 1), 舌头 (tongue; 1), 质 (tongue body; 1)
Acupoints	469	风池 (GB20; 66), 太阳穴 (EX-HN5; 51), 肩井 (GB21; 40), 大椎 (DU14; 30), 环跳 (GB30; 27), 肩髃 (LI15; 14), 少海 (HT3; 12), 委中 (BL40; 11), 承扶 (BL36; 11), 天宗 (SI11; 10)
Zang organ	139	心 (heart; 125), 肺 (lung; 5), 肾 (kidney; 4), 脾 (spleen; 3)
Meridians and collaterals	34	膀胱经 (bladder meridian, BL; 8), 胃经 (stomach meridian, ST; 6), 大肠经 (large intestine meridian, LI; 4), 肝经 (liver meridian, LI; 2), 足太阳 (bladder meridian, BL; 2), 心经 (heart meridian, HT; 1), 肺经 (lung meridian, LU; 1), 手阳明经 (large intestine meridian, LI; 1), 足少阳 (gallbladder meridian, GB; 1), 小肠经 (small intestine meridian, SI; 1)
Fu organ	3	胆 (gallbladder; 2), 胃 (stomach; 1)
Both tongue body and coating	793	舌 (tongue; 793)
Tongue coating manifestation	10,911	薄 (thin; 3612), 黄 (yellow; 1907), 腻 (slimy; 1725), 干 (dry; 791), 白 (738; white), 略黄 (slightly yellow; 570), 少 (less; 365), 厚 (thick; 254), 润 (moist; 233), 滑 (slippery; 150)
Tongue body manifestation	4088	红 (red; 893), 淡 (pale; 564), 略红 (slightly red; 467), 暗 (dark; 216), 略暗 (slightly dark; 216), 红暗 (red and dark; 195), 齿印 (teeth-marked; 144), 暗红 (dark and red; 127), 淡暗 (pale and dark; 126), 略淡 (slightly pale; 122)
Pulse condition	9573	细 (thready; 3493), 弦 (string-like; 1364), 弱 (faint; 841), 沉 (sunken; 651), 滑 (slippery; 616), 数 (534; rapid), 软 (soft; 473), 平 (normal; 420), 略弦 (slightly string-like; 180), 略数 (slightly rapid; 123)
Direction and position	5781	左 (left; 1262), 右 (right; 1110), 下 (lower; 736), 上 (upper; 282), 心 (center; 273), 中 (middle; 199), 尖 (tip; 193), 前 (front; 141), 外 (outside; 136), 外侧 (outward; 128)

#### Top-10 syndromes and their relationships with the entities of pulse and tongue body (coating) manifestations

**Fig. 6:**
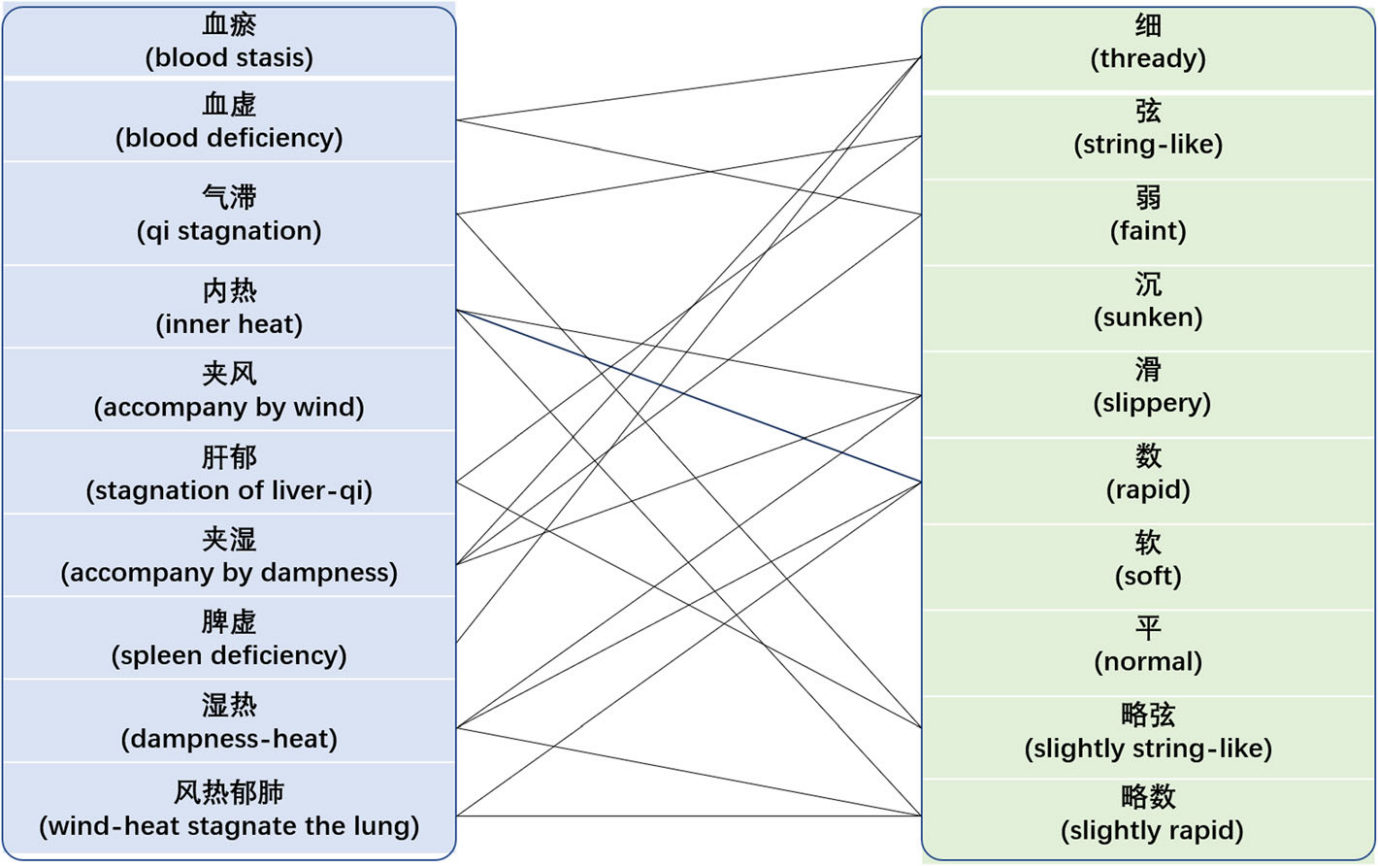
Examples of some relationships between the top-10 syndromes and top-10 pulse conditions. The solid lines suggest that there are many possible relations between syndromes and pulse conditions. The absence of a line does not mean there is no relation between them. The figure was generated by Mcrosoft PowerPoint 2016

**Fig. 7:**
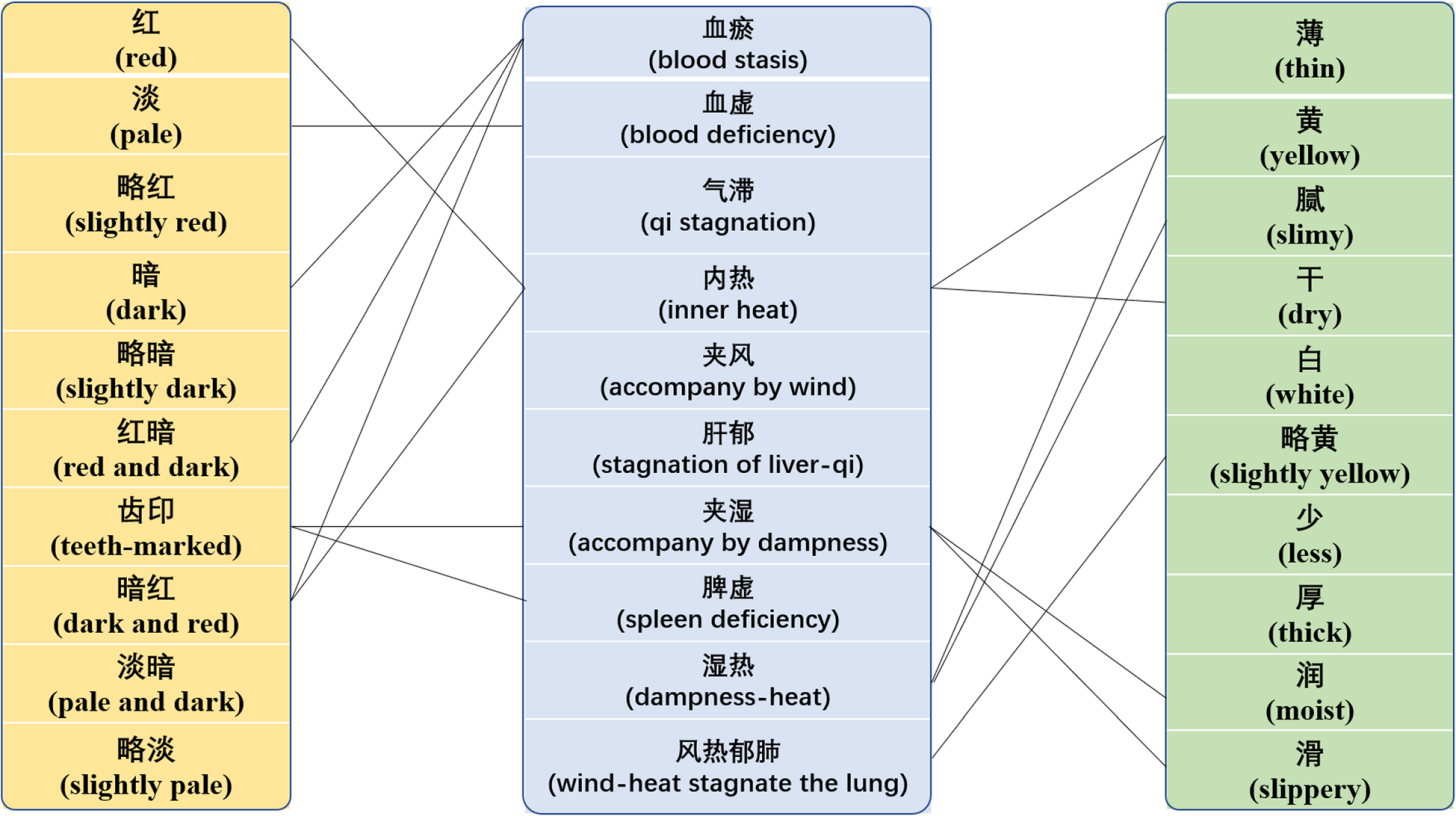
Examples of some relationships between the top-10 syndromes and top-10 tongue body and coating manifestations. The solid lines suggest that there are many possible relations between them. The absence of a line does not mean there is no relation between them. The figure was generated by Mcrosoft PowerPoint 2016

The top-10 syndromes in our preprocessed database are listed in Figs. 6 and **7**. As an important part of TCM diagnosis, syndrome differentiation, which is a comprehensive analysis of symptoms and signs, has implications for determining the cause, nature, and location of the illness and the patient’s physical condition [48]. Solid lines are used to connect entities that are likely to be to syndromes according to the textbook *Diagnostics of Traditional Chinese Medicine* [47]. Figure 6 shows that there are many-to-one and one-to-many relationships between syndromes and pulse conditions. For example, a string-like pulse is probably caused by qi stagnation and liver depression, and the blood deficiency manifests as a thready or faint pulse. In Fig. 7, the blood stasis syndrome appears as multiple clinical tongue body manifestations (dark, dark and red, or red and dark), blood deficiency manifests as a pale tongue, and the yellow coating may be the result of inner heat or dampness heat. As can be seen, common pulse or tongue body (coating) manifestations show a close relationship with common blood deficiency syndromes.^7^ For instance, in the text “时感休倦,舌淡,苔薄黄,左脉弱” (feels tired from time to time, pale tongue body, thin and yellow coating, faint pulse in left hand), and “经期延后,经量少,畏寒,疲倦,思睡,舌偏淡,苔薄润,脉细” (delayed menorrhea, fear of cold, fatigue, drowsy, pale tongue body, thin and moist coating, thready pulse), after comprehensively analyzing the clinical manifestations, both syndromes of these two cases are likely to be a blood deficiency. Here, the pale tongue body and faint and thready pulse are two important indications of blood deficiency syndrome. More examples of context regarding the top-10 syndromes are listed in Additional file 2.

There are also some exceptions. For example, blood stasis is the most frequent syndrome in our dataset. In TCM basic theory, blood stasis syndrome is likely to manifest as a rough pulse, slow pulse, or tight pulse [47]. However, these three are not mentioned in the top-10 pulse conditions. To determine why, we looked up the original text and noticed that in the TCM clinical free text, patients with blood stasis syndrome may not appear as having the above-mentioned pulse conditions. For instance, in the text “脑血管动脉瘤术后,神识不清,唇干,紫暗,痰多稠黏,大便干燥,小便清,苔黄腻,舌暗红,脉缓” (postoperative cerebral vascular aneurysm, clouded in mind, dry lips, dark purple, sticky sputum, dry stool, clear urination, yellow and greasy coating, dark red tongue, slow pulse), the syndrome of this case should be summarized as blood stasis^8^ accompanied by phlegm-heat,^9^ and the postoperative cerebral vascular aneurysm and dark red tongue body reflect the stagnated blood inside the body. However, a moderate pulse is not a typical symptom of blood stasis syndrome.

It can be seen that the main content of the corpus mostly corresponds to the annotation results. Moreover, constructing a corpus helps us to obtain and analyze the content of a dataset. However, there are some cases that do not conform to this trend. This occurs because TCM is an experience-based clinical medicine, and its clinical cases are detailed and variable. Although tongue and pulse diagnoses have a certain diagnostic function, only a comprehensive analysis by the four examination methods can help a practitioner to diagnose and treat a patient accurately.

#### Examples of special entities and analysis

In our fine-grained entity corpus, there are some special annotations that need to be explained. In most cases, the general rule is that there is a modification in the direction and location words when they occur in front of a body part, such as “右下肢” (right lower limb) or “左膝关节” (left knee joint). However, there are still some particular expressions in TCM, for example, “少腹” (lower abdomen) and “小腹” (lower abdomen), which are two of the “ordinary body part” entities in TCM. In our annotation guidelines, “少” and “小” should not be annotated as “direction and position” separately. To preserve the particular expressions of TCM, entities similar to the above two cases are not split.

There are some entities with combinable attributes in TCM. For example, the record “背心怕冷” (the center of the back is sensitive to cold), “心” means the center position on the back rather than the heart viscera. In addition, the record “心虚胆怯” (timidity due to insufficiency of qi and deficiency of blood of the heart), “心” should be annotated as a Zang organ, moreover, the word“心肌” (cardiac muscle) is an anatomical concept of Western medicine, so the entity “心” (cardiac) should be annotated as an “original body part.” In above three cases, the word “心” should be annotated as a different entity type in different contexts.

Furthermore, the case records of TCM have many abbreviations and polysemy, for example, in the transcript “左尺尤” (especially in the left chi), “尺” (chi) is a brief form of “尺脉” (chi pulse), and here the word “脉” (pulse) was omitted. In another example, the word “舌” might to be annotated as “tongue body” (e.g., “舌红” (red tongue)), “tongue coating” (e.g., “舌腻” (tongue coating is slimy)), and “both tongue body and tongue coating” (e.g., “舌可” (normal tongue)) change according to different contexts.

Some special entities are annotated as the “direction and position” entity type, such as the records “下两寸处” (two cun downward) and “外侧4寸处” (4 cun sideward). Here, “cun” is a common ancient unit of length (about 3.33 cm) especially used for locating acupoints or meridians. This is quite similar to ancient Chinese medical texts.

Hence, the annotation of TCM clinical records is complicated. It is quite different from the annotation work of Western medical records performed in previous studies, and abundant TCM knowledge is necessary for the annotators to analyze the meaning of the context.

#### Examples and analysis of entity types with lower entity counts or annotation counts

As shown in Table 3, entity classes that contain only a few entities in TCM clinical records consist of “pulse” (3.6%), “tongue coating” (1.6%), “tongue body” (1.1%), “Zang organ” (0.8%), “Fu organ” (0.3%), “meridians and collaterals” (0.98%), and “both tongue body and coating” (0.3%). Entities that occur infrequently in the annotations include “acupoint” (1.3%), “Zang organ” (0.4%), “meridians and collaterals” (0.1%), “Fu organ” (0.008%), and “both tongue body and coating” (2.1%). These results can be attributed to following reasons.

First, it is easy to form inertial thinking when annotating the entity “body part,” which results the entity “Fu organ” being rarely used as an annotation result. For example, “胃” (stomach) is annotated as “Fu organ” for twice but as “ordinary body part” 1267 times. The three annotators agreed that the entity “胃” (stomach) is more likely to express an anatomical part rather than a Fu organ. It is thus clear that TCM practitioners are highly influenced by Western medicine knowledge.

In addition, the dataset in our study consists of Chinese medicine physician case records instead of acupuncture case records. Thus, the entity “acupoint” (1.3%), and “meridians and collaterals” (0.1%) account for a very small proportion in our corpus. Table 5 lists the examples of top-10 annotated acupoints and corresponding meridians. Interestingly, from it we can see that acupoints are mostly used to describe symptoms, especially symptom of pain. We can reasonably infer that the different focus of knowledge and clinical habits of TCM physicians may also lead to this result.

**Table 5:** Examples of top-10 annotated acupoints in corresponding meridians

Meridians	Annotated acupoints and number of occurrences	Examples
Lung meridian (LU)	鱼际 (LU10; 3), 云门 (LU2; 3)	“右**云门**痛” (pain in right LU2)
Large intestine meridian (LI)	肩髃 (LI15; 14), 曲池 (LI11; 9), 合谷 (LI4; 8), 臂臑 (LI14; 7), 手三里 (LI10; 5), 巨骨 (LI16; 4), 肘髎 (LI12) (1)	“左**肩髃**痛消失” (Left LI15 pain disappears)
Stomach meridian (ST)	解溪 (ST41; 5), 髀关 (ST31; 4), 气冲 (ST30; 2), 梁丘 (ST34; 2), 下关 (ST7; 2), 内庭 (ST44; 1), 足三里 (ST36; 1), 丰隆 (ST40; 1), 人迎 (ST9; 1)	“左膝, **解溪**, 坐起时痛甚” (pain in ST41 in the left knee was aggravated when sitting up)
Spleen meridian (SP)	血海 (SP10; 1), 三阴交 (SP6; 1), 大横 (SP15; 1), 腹结 (SP14; 1)	“下肢麻痹, 左, **血海**以下, 膝痛” (paralysis of left lower extremities, below the SP10, gonyalgia)
Heart meridian (HT)	少海 (HT3; 12)	“左锁骨头痛, 右**少海**痛” (pain in left collarbone and right HT3)
Small intestine meridian (SI)	天宗 (SI11; 10), 秉风 (SI12; 5), 曲垣 (SI13; 2), 肩贞 (SI9; 1), 秉风穴 (SI12; 1), 天容 (SI17; 1)	“右**天宗**痛, 右前臂蚁行感” (right SI11 ache, a sense of ant movements in the right forearm)
Bladder meridian (BL)	委中 (BL40; 11), 承扶 (BL36; 11), 白环俞 (BL30; 10), 大肠俞 (BL; 9), 承山 (BL57; 7), 秩边 (BL54; 3), 昆仑 (BL60; 2), 通天 (BL7; 2), 申脉 (BL62; 1),	“右**委中**及**承山**旁痛, 静脉阻塞” (ache in right BL40 and BL57 aside, vein occlusion)
Kidney meridian (KI)	涌泉 (KI1; 2), 太溪 (KI3; 2), 然谷 (KI2; 1)	“脚底热感, **涌泉**” (a hot sensation in the sole of the foot, KI1)
Pericardium meridian (PC)	大陵 (PC7; 1)	“痛点, 左阳池, 左少海, 右**大陵**” (ache point, in left SJ4, HT3 and right PC7)
Triple energizer meridian (TE)	阳池 (TE4; 8), 耳门 (TE21; 2), 肩髎(TE14; 2)	手关节痛, **阳池**处, 怕冷 (hand joints ache, in the position of TE4, sensitive to cold)
Gallbladder Meridian (GB)	风池 (GB20; 66), 肩井 (GB21; 40), 环跳 (GB30; 27), 居髎 (GB29; 10), 阳陵泉 (GB34; 5), 侠溪 (GB40; 1), 维道 (GB28; 1)	“现痛点, **风池**下面” (at present, the ache point is in the GB20 below)
Liver meridian (LR)	急脉 (LR12; 2), 太冲 (LR3; 2)	“便秘, 右**急脉**处痛” (constipation, pain in right LR12)
Extra point (EX)	太阳穴 (EX-HN5; 51), 太阳 (EX-HN5; 6), 外膝眼 (EX-LE5; 4), 夹脊 (EX-B2; 3), 膝眼 (EX-B6; 2), 鹤顶 (EX-LE2; 1), 颈百劳 (EX-UX8; 1), 腰眼 (EX-B6; 1)	“头痛, **太阳穴**” (headache, in the EX-HN5)
Governor vessel (GV)	大椎 (GV14; 30), 腰阳关 (GV3; 8), 长强 (GV1; 1), 风府 (GV16; 1), 前顶 (GV21; 1)	“项背强痛, 右**大椎**曲垣甚” (stiff pain in the nape and back, especially in the right GV14 and SI13)
Conception vessel (CV)	中脘 (CV12; 1), 曲骨 (CV2; 1)	“**中脘**肋胁痛, 吐白粘痰” (pain in the CV12 and costal region, spitting white and sticky phlegm)

Furthermore, from Table 3, we can see that there are not many entities related to “tongue body” (7), “tongue coating” (10), “pulse” (22), and “both tongue body and tongue coating” (2); however, they have large number of annotations (3789, 4978, 6148, 793). Hence, one can see that the expressions of “tongue body,” “tongue coating,” “pulse,” and “both tongue body and tongue coating” are relatively consistent and frequently used in TCM clinical records.

## Conclusions and future work

Corpus construction is a fundamental and indispensable task for the development of NLP techniques with the aim of discovering valuable knowledge in TCM. In this paper, we presented a method of building a fine-grained annotated entity corpus based on case records of TCM. This paper presented the detailed steps as well as the implementation, which involves data selection, draft guideline development, iterative annotations for guideline updating, consistency assessment, and corpus construction. High IAA values were achieved in our final annotation work, indicating that our approach is effective and the corpus is of high quality. The annotated data analysis revealed some interesting point and problems, indicating that the modern TCM has integrated a lot of knowledge of Western medicine; at the same time, the construction of the corpus of TCM records is still very dependent on a professional knowledge of TCM. This work lays a solid foundation for future TCM corpus construction and NER research.

There are still some inevitable shortcomings in our work; for instance, the entity types were not comprehensive enough. Because of the limitations of time, we could not complete the annotation of all existing entities in our dataset. In future, we will annotate more entity types, such as symptoms and prescriptions, to enrich the guidelines and corpus using the methods introduced in this paper. More types of TCM clinical records from different sources will also be annotated to improve the applicability of the corpus. Furthermore, based on the corpus, we will develop algorithms to support NLP techniques. Finally, deep research of the polysemy, abbreviations, relationships among entities, and the normalization of entities are the next tasks in our future work.

## Supplementary information

**Additional file 1.** Annotation guideline.

**Additional file 2.** Information regarding the top 10 syndromes (examples of context).

## Data Availability

The datasets used and analyzed during the current study are available from the corresponding author on reasonable request.

## Abbreviations

IAA: Inter-Annotator Agreement
TCM: Traditional Chinese Medicine
NER: Named Entity Recognition
EMR: Electronic Medical Record
MOH: Ministry of Health
NLP: Natural Language Processing
i2b2: Integrating Biology and the Bedside
LU: Lung meridian
LI: Large Intestine meridian
ST: Stomach meridian
SP: Spleen meridian
HT: Heart meridian
SI: Small Intestine meridian
BL: Bladder meridian
KI: Kidney meridian
PC: Pericardium meridian
TE: Triple Energizer meridian
GB: Gallbladder meridian
LR: Liver meridian
EX: Extra point
GV: Governor Vessel
CV: Conception Vessel

## Footnotes

1 In TCM, a geographical tongue is a tongue with an irregular shedding of the coating and a distinct demarcation between the shed and unshed areas so that it looks similar to a map.

2 A “string-like pulse” is a straight, long, and taut pulse, like a musical string to the touch.

3 Tongue diagnosis is an inspection of the size, shape, color, and moisture of the tongue proper and its coating. It is very helpful for TCM doctors in disease diagnosis. For example, a yellow coating is always a manifestation of inner heat.

4 Pulse diagnosis is an examination of the pulse for making a diagnosis. TCM doctors examine the pulsation of blood vessels by feeling with the fingertips. This examination involves the pulse position, pulse shape, pulse rate, and other features. A certain type of pulse indicates a particular disease

5 In TCM, Zang-Fu organs are functional concepts, which are more than just anatomical concepts. For instance, in Western medicine, “心” (heart) refers to the anatomical heart, which has the function of pumping blood, whereas in TCM, “心” (heart) refers to a Zang organ with the functions of dominating blood circulation and vessels, controlling mental and emotional activities, and sharing a paired relationship with the small intestine.

6 In TCM, an ashi point, also known as an “ouch” point, is an acupuncture point with no specific name or definite location, the site of which is determined by tenderness or other pathological responses. Generally, a point with obvious tenderness is considered to be an ashi point.

7 In TCM, a blood deficiency syndrome is any pathological change characterized by a deficiency of blood that fails to nourish organs, tissues, and meridians/channels. A pale tongue indicates the blood deficiency.

8 Blood stasis is a pathological product of blood stagnation and includes extravasated blood and sluggishly circulating blood or blood congested in a viscus, all of which may turn into pathogenic factors. These factors, blood stasis and stagnant blood, are marked by a dark red tongue with bruises and similar features.

9 Phlegm-heat is a combination of phlegm and internal heat as an entity that causes disease.
