# Supplementary material for: Constructing fine-grained entity recognition corpora based on clinical records of traditional Chinese medicine
Source: BMC Med Inform Decis Mak. 2020 Apr 6;20:64. doi: 10.1186/s12911-020-1079-2 (PMC7132896; doi:10.1186/s12911-020-1079-2)
Supplement: Supplementary file 1 — Additional file 1. Annotation guideline. [file 12911_2020_1079_MOESM1_ESM.docx]

**Annotation guideline**

**1. “Body part”**

In TCM, “body part” can be divided into 2 categories: the “ordinary body part” and the “special body part”. Entity “ordinary body part” refers to the normal body part concepts, while “special body part” refers to the unique concept that expresses the body part in TCM.

**1.1 “Ordinary body part”**

**Definition:** This entity enables us to locate the exact positions of symptoms, medical tests, or disease. If an entity that expresses part of the body cannot be annotated as “special body part”, it will be annotated as “ordinary body part”.

**Annotation example:**

(1) “背心怕冷”(the center of back are afraid of cold), “前阴发疹”(fore pudendal rash), “下肢肿胀”(swelling of lower limbs), the annotations should be “**背[ordinary body part]**心怕冷,” “前**阴[ordinary body part]**发疹,” “下**肢[ordinary body part]**肿胀.”

(2) “咽喉疼痛”(sore throat), “头项胀痛”(distending pain in head and nape), “面部皮肤发红”(redness in skin of face), the annotations should be “**咽[ordinary body part]喉[ordinary body part]**疼痛,” “**头[ordinary body part] 项[ordinary body part]** 胀痛,” “**面部[ordinary body part] 皮肤[ordinary body part]** 发红”.

(3) Special expression of body parts in TCM: “右少腹胀”(right lower abdomen distension), “心下痞满” (epigastrium distension). Here “少腹(lower abdomen)” and “心下(epigastrium)” are expressions of body part in TCM, so the annotations are **“**右**少腹[ordinary body part]**胀” and “**心下[ordinary body part]**痞满.”

**1.2 “Special body part”**

**Definition:**

The “special body part” category contains entity “tongue body,” “tongue coating,” “pulse,” “Zang-organs,” “Fu-organs,” “meridian and collateral,” “acupoint,” **“**both the tongue body and tongue coating”. Zang-Fu organs are functional concepts, which are more than just anatomical concepts. For instance, in Western medicine, “肺”(heart) refers to the anatomical lung, which has the function of gas exchange, whereas in TCM, “肺”(heart) refers to a Zang organ with the functions of dominating blood circulation and vessels, all vessels converging in lung, and sharing a paired relationship with the large intestine. Each entity and the corresponding definitions and examples are as follows

**1) “Pulse”**

**Definition:** Radial artery of the wrist, which includes three sections: *cun*, *guan*, and *chi*. The pulse entity is annotated only when it is followed by a description of the pulse condition.

**Annotation examples:**

1. “脉弦细” (thready and string-like pulse) should be annotated as “**脉**[**pulse]** 弦细.”
2. “脉浮”(floating pulse) should be annotated as “**脉[pulse]** 浮.”

**2) “Tongue body”**

**Definition:** This is the musculature and vascular tissue of the tongue, also referring to the tongue substance. It is annotated only when followed by a specific description of the tongue’s physical manifestation.

**Annotation examples:**

1. “舌红”(red tongue body) should be annotated as “**舌[tongue body]** 红.”
2. “舌黯”( dark tongue) should be annotated as “**舌[tongue body]** 黯.”

**3) “Tongue coating”**

**Definition:** A layer of moss-like material covering the tongue, also called tongue fur. It is annotated only when followed by a specific description of tongue coating manifestation. The tongue coating is not existing in everyone, but it indeed belongs to a part of human body.

**Annotation examples:**

1. “苔白润”(white and moist tongue coating) should be annotated as “**苔[tongue coating]**白润.”
2. “舌苔厚”(thick coating) should be annotated as “**舌苔[tongue coating]** 厚.”

**Attention:** These entities (“pulse”, “tongue body” and “tongue coating”) we need to mark are the Chinese word related to pulse diagnosis and tongue diagnosis, not the symptoms related to tongue or pulse. For instance, “舌痛”(tongue pain) is a symptom rather than tongue diagnosis, and “舌” should be annotated as “**body part**” instead of “tongue body.”

**4) “Both tongue body and tongue coating”**

**Definition:** It is the words referring to both the tongue body and tongue coating. In the clinical records of TCM, if the tongue coating and tongue body have no positive signs need to record or no change compared to last visit, it is usually carried out in brief, TCM clinical practitioners will describe the body and coating of the tongue with one word.

**Annotation examples:**

1. “舌可”(normal tongue) should be annotated as “**舌[both tongue body and tongue coating]**可.”
2. “舌正”(normal tongue) should be annotated as “**舌[both tongue body and tongue coating]** 正.”
3. “舌如前”(tongue body and tongue coating are same as last visit) should be annotated as “**舌[both tongue body and tongue coating]** 如前.”

**5) Zang organ,**

**Definition:** An internal organ in which the essence and qi are formed and stored. These organs include heart, liver, spleen, lungs, and kidneys, and are also called the five viscera.

**Annotation examples:**

“见肝之病，知肝传脾”(If there is a disease in the liver, it is known that the liver disease may be transmitted to the spleen): 肝(liver) and 脾(spleen) belongs to five Zang-organs, so the annotations are “见 **肝[Zang organ]** 之病，知 **肝[Zang organ]** 传 **脾[Zang organ]**.”

**6) Fu organ**

**Definition:** An internal organ in which food is received, transported, and digested, including the gallbladder, stomach, large intestine, small intestine, urinary bladder, and triple energizers. They are also called the six bowels.

**Annotation example:**

“疏肝利胆” (dispersing stagnated liver qi for promoting bile flow), “胆”(the gallbladder) should be annotated as “疏肝利 **胆[Fu organ]**.”

**Attention:**

Zang-Fu organ we need to mark are the Chinese words originated from viscera theory, they are functional concepts rather than anatomical organs.

**7) “Acupoint”**

**Definition:** Point where a needle is inserted and manipulated in acupuncture therapy. In TCM, acupoint is a functional concept with no visible form. In clinical, acupoints in twelve regular meridians and eight extra channels, *ashi* point, are commonly used for diagnosis and treatment. For example, unusual pain, knot or other changes at an acupoint are sometimes signs which indicate a disease.

**Annotation example**

1. “肩髃穴疼痛” (pain in LI15) should be annotated as “**肩髃穴[acupoint]**疼痛.”
2. “右太阳穴痛” (pain in the right EX-HN5) should be annotated as “右**太阳穴[acupoint]**痛.”

**8) Meridian and collateral**

**Definition:** A system of conduits through which qi and blood circulate, connecting the bowels, viscera, extremities, superficial organs, and tissues, and making the body an organic whole. These are the same as channels and networks and are also called meridians or channels. For instance, stomach meridians connect with head and lower limbs externally and stomach Fu organ internally. Therefore, the acupoints (e.g. ST 36) on the Stomach meridian are usually used to treat the stomach disease.

**Annotation examples:**

1. “臂痛,左大肠经(large intestinal meridian)” should be annotated as “臂痛,左**大肠经[meridian and collateral]**.”
2. “右上肢痛,手阳明”(right upper limb pain in the hand *Yangming* meridian) should be annotated as “上肢痛,**手阳明[meridian and collateral]**,右甚.”

**2. Direction and position**

**Definition:** Description of the direction and position, which enables us to know the specific location of the body part. The direction and position descriptions involves “左和右”(left and right), “前和后”(front and back), “上和下”(above and below), “外和内”(inside and outside), “桡侧和尺侧”(radialis and ulnaris), “腹侧和背侧”(ventral and dorsal), etc.

**Annotation examples:**

1. “右少腹胀” (distension feeling in right abdomen) should be annotated as “**右[direction and position]**少腹胀.”
2. “左侧腰痛” (left low back pain) should be annotated as “**左侧[direction and position]**腰痛.”
3. “腰以下发热” (hot feeling below waist) should be annotated as “腰**以下[direction and position]**.”
4. “舌上满布裂纹” (the tongue is covered with fissures) should be annotated as “舌**上[direction and position]**满布裂纹.”
5. “背心怕冷(back center is afraid of cold)”, “心” means the center position on the back other than the heart viscera, it should be annotated as “背**心[direction and position]**怕冷.”
6. “苔中根部白”(the middle and root of the tongue coating is white) should be annotated as “苔**中[ direction and position] 根部[direction and position]**白.”
7. “舌尖红”(red tip of tongue) should be annotated as “舌**尖[direction and position]**红.”
8. “左脉弦”(string-like pulse in the left hand) and “右尺脉不足” (insufficient *chi* pulse manifestation in the right hand) should be annotated as “**左[direction and position]**脉弦” and “**右[direction and position]**尺脉不足.”

**Attention:**

1. The “direction and position” may be the combination of more than two elements. For example, “右下肢桡侧(radialis of right lower limb)”. After discussion, it should be annotated as “**右[direction and position] 下[direction and position]** 肢 **桡侧[direction and position].”**
2. “上下楼疼痛”(feel pain when up and down the stairs), here “上”(up) and “下”(down) are not expressions of “direction or location”. So, there is no need to annotate.

**3. “Tongue body manifestation”**

**Definition:** It refers to the description of the tongue body in TCM, relating to changes in the color and form of the tongue body, including size, shape, color and moisture of

the tongue proper.

**Annotation examples:**

1. “舌淡红”(light red tongue): “淡红”(pink) is uses to describe the tongue color, “淡”(light) describe the degree of the red color. So the annotation is “舌**淡红[tongue body manifestation].**”
2. “舌淡胖”(pale and enlarged tongue): “淡”(pale) means the pale color of tongue, “胖”(enlarged) refers to the tongue is larger than normal. The complete expression should be “舌淡舌胖”(pale tongue and enlarged tongue), but according to the Chinese linguistic habit, the second “舌”(tongue) is always omitted, So the annotations are “舌**淡[tongue body manifestation]胖[tongue body manifestation]**.”

**4. “Tongue coating manifestation”**

**Definition:** It is the description of the color and the texture of the coat, the texture includes thickness, moisture, sliminess and roughness etc. the color involves white, yellow, gray, black, and in rare cases, green.

**Annotation examples:**

1. “苔黄褐色,中心略厚” (yellowish-brown coating, a litter bit thick in the center), “黄褐色”(yellowish-brown) refers to the coating color, “略厚” (a litter bit thick) refers to the thickness of the coating texture. So the annotations are “苔**黄褐色[tongue coating manifestation]**, 中心**略厚[tongue coating manifestation]**.”
2. “苔薄少津”(thin coating with less fluid), “薄”(thin) and “少津”(less fluid) both refer to the coating nature, so the annotations are “苔**薄[tongue coating manifestation] 少津[tongue coating manifestation]**.”
3. “舌黄腻” (slimy and yellow coating on the tongue): here the Chinese character “舌(literal meaning: tongue)” express the coating on the tongue. So the annotations are “舌**黄[tongue coating manifestation] 腻[tongue coating manifestation]**.”
4. “舌苔黄” (yellow tongue coating) should be annotated as “舌苔**黄[tongue coating manifestation]**.”

**5. “Pulse condition”**

**Definition:** Pulse condition is the description of arterial pulsation in TCM by feeling with the fingertips. It contains position, frequency, shape, tendency and rhythm of the pulse. The pathological pulse condition contains floating, sinking, slippery, slow, string-like, weak, gaseous, intermittent, etc.

**Annotation examples:**

1. “脉弦” (string-like pulse) should be annotated as “脉**弦[Pulse condition]**.”
2. “脉弦滑” (slippery and string-like pulse) should be annotated as “脉**弦[pulse condition] 细[pulse condition]**.”

**Supplementary specification**

1. There are some polysemy phenomena in Chinese records, the annotations should be done with understanding the context. For example, “心肌” (cardiac muscle), “心虚胆怯” (timidity due to insufficiency of qi and deficiency of blood of the heart), “手心” (hand center), “心下痛” (epigastrium pain) all have the Chinese character “心,” but they have different meanings. The “心” in “心肌” refers to the anatomical heart, the “心” in “心虚胆怯” refers to the heart of the five Zang organs in TCM, the “心” in “手心” express the center of the hand, the combination of “心” and “下” is an expression of body part in TCM which means epigastrium. So the annotations are “**心[body part]** 肌,” “**心[Zang organ]** 虚胆怯,” “手**心[direction and position)],**” “**心下[body part]** 痛.”
2. There are some English word abbreviation in clinical texts, such as “C5,6椎间盘脱出”(herniated disc of fifth and sixth cervical vertebrae), “C” is the abbreviation of cervical vertebra in medical records, so the annotations are “**C5,6[body part]**椎间盘脱出”.
3. The sentence with missing elements, for example “左浮”(left floating), the complete expression should be “左脉浮”(left pulse floating)，here the word “脉” was omitted, and the annotations are “**左[direction and position] 浮[pulse condition]**”.
4. There is no need to mark the words without clinical medical significance, for example, “尿血,肉眼没有” (hematuria but not visible), here “肉眼” has no practical medical significance and will not be annotated.
