# Supplementary material for: Constructing fine-grained entity recognition corpora based on clinical records of traditional Chinese medicine
Source: BMC Med Inform Decis Mak. 2020 Apr 6;20:64. doi: 10.1186/s12911-020-1079-2 (PMC7132896; doi:10.1186/s12911-020-1079-2)
Supplement: Supplementary file 2 — Additional file 2. Information regarding the top 10 syndromes (examples of context). [file 12911_2020_1079_MOESM2_ESM.docx]

**Information regarding the top 10 syndromes (examples of context)**

**1. 血瘀 (Blood stasis)**

e.g. “经色暗红, 颈背强痛, 白带粘稠, 失眠, 体倦, 手足心热, 口干, 舌淡红略暗瘀点, 脉细.” (Dark red menstruation, strong pain in neck and back, sticky leucorrhea, insomnia, fatigued, feverish palms and soles, dry mouth, slightly dark red tongue with stasis point, fine pulse.)

In this example, dark red menstruation and dark red tongue with stasis point are two important indications of blood deficiency syndrome.

**2. 血虚 (Blood deficiency)**

e.g. “头昏身软, 气短乏力, 声低, 懒言, 不思饮食, 面黄, 怕冷, 汗多, 睡眠差, 苔白腻, 舌淡, 脉弦, 重按无力.” (Dizziness and faint body, shortness of breath, low voice, lazy to talk, no appetite, sallow complexion, afraid of cold, sweaty, poor sleep, white and greasy coating, pale tongue, string-like pulse, powerless pulse when taking deeply.)

In TCM, blood deficiency is syndrome marked by pale or sallow complexion, pale lips and nails, dizziness, dimmed vision, palpitations, numbness of extremities and fine pulse. In this example, the symptoms, dizziness, faint body, faint body, pale tongue reflect the deficient blood inside the body.

**3. 气滞 (Qi stagnation)**

e.g. “气喘, 咳嗽, 紧张, 情绪抑郁, 眠差, 易醒, 纳差, 肢冷, 喜暖, 苔黄略腻, 脉弦细数.” (shortness of breath, cough, nervousness, depression, poor sleep, easy to wake up, poor appetite, cold limbs, fond of worm, yellow and slightly greasy coating, the pulse is string-like, fine and rapid.)

In TCM, qi is an essential part of which constitutes the body and maintain the activities of life, visceral function and metabolism. Depression is an important cause of qi stagnation. In this example, depression and sting-like pulse are two important manifestations of qi stagnation syndrome.

**4. 内热 (Inner heat)**

e.g. “身热, 汗多, 头面甚, 大便燥结, 口渴, 饮多, 尿多, 次多量多, 尿急, 尿频面赤如醉状, 脚冷, 舌红, 苔薄略腻, 右脉滑, 关尤.” (Hot body, sweaty especially on the head and face, constipation, thirst, fond of drink, diuresis, frequent and urgent urination, flushed face like drunk, cold feet, red tongue, thin and slightly greasy coating, the pulse is slippery especially on right *guan*.)

In this case, inner heat, as a pathogenic factor characterized by the heat that is apt to injure fluid (e.g. constipation). The symptoms, hot, sweaty, red tongue, slippery pulse and other features reflect the inner heat syndrome.

**5. 夹风 (Accompany by wind)**

e.g. “皮肤红疹, 瘙痒, 热明显, 晚上口渴, 饮水, 大便先干, 苔薄黄, 舌质红, 脉细.” (Red rash, itches, hot body, thirst at night, fond of drink, dry stool, thin and yellow coating, red tongue, fine pulse.)

In this example, red rash with itches is caused heat syndrome with pathogenic wind.

**6. 肝郁 (Stagnation of liver-qi)**

e.g. “月经量多, 无痛经, 经前情绪不好, 腹胀, 经期加重, 白带正常, 口干, 时身热, 眠欠安, 舌略红, 苔薄黄, 脉细弦.” (More menstruation, no dysmenorrhea, depression before menstruation, abdominal distension which is aggravated during menstrual period, normal leucorrhea, dry mouth, hot body at times, poor sleep, the tongue is slightly red with thin and yellow coating, fine and string-like pulse.)

In TCM, the stagnation of liver-qi a syndrome marked by depression, frequent sighing, hypochondriac or lower abdominal distention or moving pain, and string-like pulse; and in women, distending pain of the breast and irregular menstruation, the same as the liver qi stagnation syndrome. In this example, depression, abdominal distension and string-like pulse are three important manifestations of stagnation of liver-qi syndrome.

**7. 夹湿 (Accompany by dampness) and 脾虚 (spleen deficiency)**

e.g. “大便溏, 日一次, 小便不黄, 口苦, 不思饮, 苔薄黄, 舌有齿痕, 淡胖.” (Loose stools once a day, urine is not yellow, bitter mouth, no thirst for water, thin and yellow coating, the tongue is enlarged and teeth-marked with pale color.)

According to the theory of TCM, dampness contains internal dampness and external dampness. Spleen deficiency causes internal dampness. In this case, loose stool, enlarged and teeth-marked tongue with pale color reflect the deficient spleen accompanied by dampness.

**8. 湿热 (Dampness-heat)**

e.g. “五天未便, 腹胀, 口味好, 腹痛, 面色淡黄, 睡眠多, 不能吃燥热食物, 不易感冒, 舌胖, 苔黄腻, 舌暗红, 脉微细.” (No stool for five days, abdominal distention, good appetite, abdominal pain, slightly yellow complexion, somnolence, averseness of hot food, not easy to catch cold, enlarged and dark red tongue, yellow and greasy coating, faint and fine pulse.)

In this example, yellow and greasy coating is the symptom of most diagnostic value.

**9. 风热郁肺 (Wind-heat stagnate the lung)**

e.g. “头昏, 咳嗽, 吐黄痰, 咳声不扬, 发热, 流清涕, 咽红不痛, 口渴, 苔薄, 舌红, 脉浮数.” (Mental confusion, cough, spit yellow phlegm, silent cough, fever, running nose, red pharynx without pain, thirst, thin coating, red tongue, floating and rapid pulse.)

In TCM, the syndrome of common cold is generally divided into wind-heat and wind-cold. The example is a medical record of wind-heat syndrome. The symptoms of red pharynx, thirst, running nose, floating and rapid pulse and other features are the diagnostic basis of common cold of wind-heat syndrome.
